# Applying Item Response Theory (IRT) Modeling to an Observational Measure of Childhood Pragmatics: The Pragmatics Observational Measure-2

**DOI:** 10.3389/fpsyg.2019.00408

**Published:** 2019-02-28

**Authors:** Reinie Cordier, Natalie Munro, Sarah Wilkes-Gillan, Renée Speyer, Lauren Parsons, Annette Joosten

**Affiliations:** ^1^School of Occupational Therapy, Social Work and Speech Pathology, Faculty of Health Sciences, Curtin University, Perth, WA, Australia; ^2^Discipline of Speech Pathology, Faculty of Health Sciences, The University of Sydney, Sydney, NSW, Australia; ^3^School of Allied Health, Faculty of Health Sciences, Australian Catholic University, Sydney, NSW, Australia; ^4^Department of Special Needs Education, University of Oslo, Oslo, Norway; ^5^Department of Otorhinolaryngology and Head and Neck Surgery, Leiden University Medical Center, Leiden, Netherlands

**Keywords:** pragmatic language, Rasch analysis, children, psychometrics, observational measure

## Abstract

Assessment of pragmatic language abilities of children is important across a number of childhood developmental disorders including ADHD, language impairment and Autism Spectrum Disorder. The Pragmatics Observational Measure (POM) was developed to investigate children’s pragmatic skills during play in a peer–peer interaction. To date, classic test theory methodology has reported good psychometric properties for this measure, but the POM has yet to be evaluated using item response theory. The aim of this study was to evaluate the POM using Rasch analysis. Person and item fit statistics, response scale, dimensionality of the scale and differential item functioning were investigated. Participants included 342 children aged 5–11 years from New Zealand; 108 children with ADHD were playing with 108 typically developing peers and 126 typically developing age, sex and ethnic matched peers played in dyads in the control group. Video footage of this interaction was recorded and later analyzed by an independent rater unknown to the children using the POM. Rasch analysis revealed that the rating scale was ordered and used appropriately. The overall person (0.97) and item (0.99) reliability was excellent. Fit statistics for four individual items were outside acceptable parameters and were removed. The dimensionality of the measure showed two distinct elements (verbal and non-verbal pragmatic language) of a unidimensional construct. These findings have led to a revision of the first edition of POM, now called the POM-2. Further empirical work investigating the responsiveness of the POM-2 and its utility in identifying pragmatic language impairments in other childhood developmental disorders is recommended.

## Introduction

No matter how much one desires a solitary existence or an expansive network of friends, we all engage in social interaction. For school-aged children, peer–peer interaction during play is a major context for social interaction (Cordier et al., 2013). Crucially, this context allows children to develop, express and refine pragmatic language skills. Pragmatic language has been defined as “behavior that encompasses social, emotional, and communicative aspects of social language” (Adams et al., 2005, p. 568). Pragmatic language difficulties (PLD) are implicated in various clinical populations, including autism spectrum disorder (ASD) and Attention Deficit Hyperactive Disorder (ADHD) (Young et al., 2005; Staikova et al., 2013), and is a key diagnostic feature of social (pragmatic) communication disorder (SCD) in the DSM-5 (American Psychiatric Association, 2013). As such, clinicians and researchers require robust measures to plan and evaluate interventions, and to understand more about the complex nature of this language domain.

The complex and multifaceted nature of pragmatic language makes it challenging to develop instruments for meaningful measurement of the construct. The formal nature of standardized assessment tasks fails to capture that pragmatic language is context dependence; instead, capturing a narrow and possibly unreliable picture of an individual’s pragmatic “knowledge” as opposed to performance capabilities. This limitation was accentuated when a proportion of children with SCD demonstrated knowledge of pragmatic rules, but still violated the same rules during social interactions with a communication partner (Lockton et al., 2016). Parent report measures can provide an understanding of a child’s abilities across various social contexts and have a role in understanding a child’s needs from the perspective of the service-user (Adams et al., 2012). However, if used to evaluate intervention effectiveness, they introduce bias due to the inability to blind parents to treatments.

Although observational measures show promise in addressing these known biases, there are very few in existence and the strength of their psychometric properties remains largely unknown (Adams, 2002). Furthermore, there is a need to develop pragmatic assessments that capture interactions in a naturalistic context, rather than focused on the impairment level. A recent international consensus study for Developmental Language Disorder (DLD) made specific recommendations in this regard (Bishop et al., 2016, 2017). This is especially important in the measurement of pragmatic language, as it is an area with a dearth of developmental norms (Adams, 2002).

To observe and measure pragmatic language behaviors in a functional childhood activity (play), the Pragmatics Observational Measure (POM) (Cordier et al., 2014) was developed. This instrument rates 27 items that reflect five elements of verbal and non-verbal pragmatic language: (1) introducing and responding to peer–peer social interactions; (2) use and interpretation of non-verbal communication; (3) social-emotional attunement of one’s own thinking, emotions and behavior, as well as the intention and reactions of peers; (4) higher level thinking and planning; and (5) peer–peer negotiation skills including suggestions, cooperation and effective disagreement. Children in peer–peer interactions during free and uninterrupted play are videoed and then rated according to these five elements with individual items scored along a four-point scale. To date, the POM has been used to investigate the pragmatic language abilities of typically developing school-aged children and their peers diagnosed with ADHD (Cordier et al., 2017b; Wilkes-Gillan et al., 2017a,b) and Autism Spectrum Disorder (Parsons et al., unpublished; Parsons et al., 2018).

Evaluating an instrument’s psychometric properties is an important element of test development. The POM has demonstrated good reliability, validity, responsiveness and interpretability (Cordier et al., 2014). In terms of internal consistency, an exploratory PC and a confirmatory Maximum Likelihood (ML) were performed. Factor analysis identified that the items on the POM reflected a unidimensional construct accounting for 81.5% of the variance (exploratory PC factor analysis) and 73.7% (ML factor analysis). This suggests that while items are theoretically grouped under five elements of pragmatic language, the instrument’s items represent overall pragmatic language ability rather than multidimensional “sub-dimensions” of pragmatic language. Our previous work used the Pragmatic Protocol (Prutting and Kittchner, 1987) as our “gold standard” comparison as there were very few observational assessments for assessing peer–peer pragmatic language. Labeled as a descriptive taxonomy, the Pragmatic Protocol (PP) has 30 items which are classified under verbal, paralinguistic and non-verbal aspects. Children aged 5 years and older are observed in a dyadic interaction while the rater scores each item as: appropriate, inappropriate, or not observed. While theoretically and clinically motivated, it is not clear whether this instrument reflects an underlying uni- or multidimensional construct. Thus far, the psychometric analysis of the POM was based on Classical Test Theory (CTT) approaches which view the whole test as the unit of analysis and assume that all items are equally contributing to the same underlying construct (Cordier et al., 2014). This could be problematic for the POM given that this measure has 27 items and we cannot be completely confident that all items equally contribute. There is also the possibility that all items do not reflect the same underlying construct. To explore these notions further we turned to item response theory (IRT).

Item response theory (IRT) modeling has become an important methodology for test development (Edelen and Reeve, 2007). Item response theory examines the reliability of each item and whether each item contributes to an overall construct (Linacre, 2016a). Another advantage is that IRT can be completed independently from the testing group used. Rasch analysis – a type of IRT model – has been used to successfully critique and evaluate existing measures in other clinical areas relating to swallowing and communication disorders (Donovan et al., 2006; Cordier et al., 2017a). In this study, we apply the Rasch measurement model to further evaluate the POM. Specifically, we investigated person and item fit statistics, response scale, dimensionality of the scale, and differential item functioning.

## Materials and Methods

### Participants

Video footage of children and their playmates from Cordier et al. (2010) was used to evaluate the psychometric properties of the POM. The sample included children diagnosed with ADHD (*n* = 108), paired with typically developing playmates (*n* = 108), with one child with ADHD and one typically developing child in each observation. Children with ADHD were chosen because of their known social impairments and difficulties with pragmatic language (Staikova et al., 2013). The control group involved two typically developing children in each observation (*n* = 126). Children in the control group were matched on age, sex and ethnicity and not known to have ADHD as defined by the DSM-IV (American Psychiatric Association, 2000). All playmate pairs were familiar with each other.

#### Children With ADHD

Children with ADHD were recruited from district health boards and pediatric practices in Auckland, New Zealand. The inclusion criteria detailed that children must have a formal diagnosis of ADHD from a psychiatrist or pediatrician according to the DSM-IV criteria. Children must not have been administered medication prescribed for ADHD on the day of assessment and must not have been taking medication where an overnight period was an insufficient wash-out (e.g., Atomoxetine). This was applied to ensure high levels of diagnostic accuracy, minimize inclusion of borderline cases and cases with disorders other than ADHD as the primary diagnosis, and to enable observation of how children with ADHD interact without the effects of medication.

### Typically Developing Children in the Control Group

Children in the control group (*n* = 126) were recruited from professional networks such as local schools and families of health service employees in Auckland, New Zealand. The inclusion criteria for the control group defined a typically developing child as a child with no childhood developmental disorder and no developmental concerns having been raised by a teacher or health professional. Presence of a developmental disorder was further ruled out through the administration of the Conners’ Parent Rating Scales-Revised [CPRS-R]. The CPRS-R is a screening questionnaire completed by parents or primary carers to determine whether children aged 3–17 years have signs and symptoms consistent with a diagnosis of ADHD. Previously, the CPRS-R has shown excellent reliability (international consistency reliability 0.75–0.94) and construct validity (to discriminate ADHD from the non-clinical group: sensitivity 92%, specificity 91%, positive predictive power 94%, negative predictive power 92%) (Conners et al., 1998; Conners, 2004). Children who scored below the clinical cut-offs for any CPRS-R subscale and DSM-IV subscale were included in the control group.

### Instruments

#### Pragmatics Observational Measure (POM)

The POM was developed as a result of the need for an observational measure to assess pragmatic language in naturalistic contexts between peers. Only the Pragmatic Protocol (PP) (Prutting and Kittchner, 1987) and the Structured Multidimensional Assessment Profiles (S-MAPs) (Wiig et al., 2004) were found to be observational measures of some aspects of pragmatic language. The S-MAPs was developed as a tool for clinicians for curriculum-based assessment and intervention for children and provided clinicians with examples of how to develop their own rubrics and included a few rubrics related to aspects of pragmatic language. However, this measures usefulness within a research setting is limited, as little psychometric information has been published. The PP presents similar issues, with limited psychometric information available. Moreover, the use of a dichotomous rating scale limits the observer’s ability to capture the complex nature of pragmatic language. A summary of the five elements and a summative description of each item that are grouped within each of the five elements are provided in Table 1.

**Table 1:** Pragmatics observational measure element and item description.

POM items	Summative item description
**Element: introduction and responsiveness**	
(1) Select and introduce	Selects and introduces a range of conversational topics
(2) Maintain and change	Maintains and changes conversational topics appropriately
(3) Contingency	Shares or adds information to the previously communicated content
(4) Initiate	Initiates verbal communication appropriate to the context
(5) Respond	Responds to communication given by another
(6) Repair and review	Repairs and reviews conversation when a breakdown in communication occurs
**Element: non-verbal communication**	
(7) Facial expression	Uses and responds to a variety of facial expressions to express consistent meanings
(8) Gestures	Uses and responds to identifiable, clear, intentional body actions or movements
(9) Body posture	Uses and responds to clear, identifiable body positioning and stance
(10) Distance	Use of physical space between speakers
**Element: social-emotional attunement*^§^***	
(11) Emotional attunement	Being aware of and responsive to another’s emotional needs
(12) Self-regulation	Regulate own thinking, emotions and behaviors
(13) Perspective taking	Considers/integrates another’s viewpoint/emotion
(14) Integrating communicative aspects	Appropriate use of social language within context
(15) Environmental demands	Adapts behavior to environmental demands
**Element: executive function**	
(16) Attention, planning, initiation	Attends to communicative content, plans and initiates appropriate responses
(17) Communication content	Interprets, plans, organizes and delivers content
(18) Creativity	Versatile ways to interpret/connect/express ideas
(19) Thinking style	Thinks and articulates abstract and complex ideas
**Element: negotiation**	
(20) Conflict resolution	Uses appropriate methods for resolving disagreement
(21) Cooperation	Works together; mutually beneficial exchange
(22) Engagement/Interaction	Consistently gets along well with another peer while engaged
(23) Assertion	Makes clear own opinions, viewpoints and emotions
(24) Express feelings	Expresses feelings appropriate to the context
(25) Suggests	Makes suggestions and offers opinions
(26) Disagrees	Disagrees in an effective way that promotes the interaction
(27) Requests	Requests explanations/more information in an effective way

^§^*The item Discourse interruption (originally included under the element Social-emotional attunement) was removed following factor analysis.*

The 27 items included in the POM were selected, developed and refined by the first four authors. All the authors have extensive experience in working with children from four disciplines: clinical psychology, epidemiology, speech and language pathology and occupational therapy. The item level descriptors were continuously refined over an 18-month period to ensure that they were clear, unambiguous and that all items could be rated using observable behavior. External raters assisted with item refinement by rating video footage of typically developing children and children with behavioral disorders and PLD.

The POM includes 27 items. Each item is based on the child’s consistency of performance, rated on a 4-point scale (1–4) ranging between: 1 – rarely or never observed; 2 – sometimes observed (25–50% of the time); 3 – much of the time observed (50–75% of the time); and 4 – almost always observed (75–100% of the time). A detailed description is provided for each level of performance for all items. Discriminant analysis was used during initial development and psychometric testing to calculate a diagnostic cut off score of 8.02 for significant PLD (Cordier et al., 2014). Children with a mean measure score below 8.2 were classified as having significant PLD and those above the cut-off were deemed to have no PLD.

### Procedure

The Sydney University Human Ethics Research Committee provided ethical approval to perform secondary analysis on data. The original study aimed to compare the play skills of children with ADHD with typically developing children (Cordier et al., 2010). Peer–peer social interactions for all children were observed. For those in the control group, children were observed using a designated play area at the respective schools that children attended, and children with ADHD were observed at clinics that they regularly attended. The same toys were present during all play sessions and the children were allowed to choose their play materials and activities. A diversity of play materials and toys catering to age and gender differences were made available to support a range of play and encourage peer–peer interaction.

The assessor introduced the peers to the free play situation and was as unobtrusive as possible. Participants were instructed that they could play with any of the toys in the playroom for 20 min and that they should ignore the assessor who was present in the playroom. The play session was video recorded for later analysis. Children were asked to ignore the assessor present in the playroom. When children attempted to interact with the assessor, their response was neutral and the assessor remained as unobtrusive as possible. The assessor did not intervene unless a child was in danger.

A single experienced rater (who was not the assessor) rated all the children from the videotapes. The rater was blinded to the purpose of the study to minimize bias. To establish adequate inter-rater reliability, another blinded rater familiarized themselves with the POM. Next, the first and third author developed a training video using footage of school-aged children playing who were independent of the current study. The blinded rate and their author then coded ten samples from this footage using the POM. Coding was compared and then consensus reached following discussion and re-viewing of the training footage. Reliability for the current study was calculated based on a random selection of 30% of all data.

### Statistical Analysis

Rasch analyses were used to evaluate the reliability and validity of the POM. Data were analyzed using WINSTEPS version 3.92.0 (Linacre, 2016b), with the joint maximum likelihood estimation rating scale estimation (Wright and Masters, 1982). Data were analyzed for all 27 POM items, thereafter an iterative process was adopted. This involved removing poor fitting items, in various combinations, and re-running the analysis to get the best overall item fit, person separation and dimensionality statistics. The following analyses were conducted for all investigations.

#### Rating Scale Validity

Examination of the rating scale validity can confirm whether the ordinal response scale for all items stays true to the assumption that higher ratings indicate “more” and lower ratings indicate “less” of the concept under assessment. In WINSTEPS, rating scale response options are referred to as *categories*. There are three situations in which the partial credit model can be used: (1) items where some responses may be more correct than others; (2) items that can be broken down into component tasks; and (3) items where increments in the quality of performance are rated (Wright, 1998). None of these situations apply to the POM scale structure and all POM items have the same scale structure. As such, a Rating Scale Model (RSM) was used. In alignment with the POM response options the categories are numbered 1–4.

To determine if the rating response scales were being used in the expected manner, category response data was examined for even distribution or category disorder. Poorly defined categories or the inclusion of items that do not measure the construct result in non-uniformity/disordering. Ordered categories are indicated by average measure scores (frequency of use) that increase monotonically as the category increased. Mean squares (MnSq) outside 0.7–1.4 indicate category misfit and disordering and the collapsing of the misfitting category with an adjacent category should be considered (Linacre, 2016a).

The point at which there is equal probability of a response in either of two adjacent categories being selected, known as step calibrations or Andrich-thresholds, were determined to assess step disordering. Andrich-thresholds reflect the distance between categories and should progress monotonically, showing neither overlap between categories nor too large a gap between categories. Step disordering indicates that the category defines a narrow section of the variable but does not imply that the category definitions are out of sequence. The average measure distinct categories are indicated by an increase of at least 1.0 logit on a 5-category scale. An increase of >5.0 logits, however, is indicative of gaps in the variable (Linacre, 1999).

#### Person and Item Fit Statistics

Construct validity was assessed using fit statistics to identify misfitting items and the pattern of responses for each person. Fit statistics are reported as log odd units (logits) and indicate whether the items contribute to the one construct (i.e., pragmatic language ability) and the degree to which a person’s responses are reliable. Unstandardized MnSq or Z-Standard (Z-STD) scores can be used to described infit and outfit MnSq values should be close to 1.0 with an acceptable range of 0.7–1.4 (Bond and Fox, 2015). The outfit Z-STD values are expected to be 0 and any value that exceeds ±2 is interpreted as less than the expected fit to the model (Bond and Fox, 2015). Model underfit degrades the model and requires further investigation to determine the reason for the underfit. Model overfit, on the other hand, does not always degrade the model but still can lead to the misinterpretation that the model worked better than expected (Bond and Fox, 2015).

Internal consistency of the measure is evaluated through the person reliability, which is equivalent to the traditional Cronbach’s alpha. Low person reliability values (<0.8) indicate having too few items or a narrow range of person measures (i.e., not having enough persons with more extreme abilities, both high and low).

If outlying measures are accidental, people are classified using person separation. However, if the outlying measures represent true performances, people are classified using person separation index (PSI)/strata (4^∗^person separation +1/3). To distinguish high performers from low performers, person separation determines whether the test separates the sample into sufficient levels. Low person separation is indicative that the measure is not sensitive enough to separate low and high performers. Reliability of 0.5, 0.8, and 0.9, respectively, indicates separation into only one or two levels, 2–3 levels, and 3–4 levels (Linacre, 2016a). A PSI/strata of 3 is required (the minimum level to attain a reliability of 0.9) to consistently identify three levels of performance. Item hierarchy with <3 levels (high, medium, low) is verified by item reliability. If item reliability < 0.9, the sample is too small to confirm the construct validity (item difficulty) of the measure.

#### Dimensionality of the Scale

Dimensionality can be assessed by the following means: (a) using negative point-biserial correlations identify any potentially problematic items; (b) identifying misfitting persons or items using Rasch fit indicators; and (c) performing Rasch factor analysis using Principal Component Analysis (PCA) of the standardized residuals (Linacre, 1998). The number of principal components are checked using PCA of residuals to confirm that there are no second or further dimensions after the intended or Rasch dimension is removed. No second dimension is indicated if the residuals for pairs of items are uncorrelated and normally distributed. The following recommended criteria are used to determine if further dimensions in the residuals are present: (a) the Rasch factor uses a cut-off of >60% of the explained variance; (b) on first contrast the eigenvalue of <3 (equivalent to three items), and (c) first contrast of <10% of explained variance (Linacre, 2016a).

The person–item dimensionality map using a logit scale schematically represents the distributions of the person abilities and item difficulties. In this paper, person ability refers to the level of pragmatic language ability observed by an assessor. “Difficult” items on the POM would attempt to capture aspects of pragmatic language that occurs with such infrequency that very few assessors will give a high rating to these items, whereas “easy” items might refer to aspects of pragmatic language that occurs regularly and will receive high assessors’ ratings. If two or more items represent similar difficulty, these items occupy the same location on the logit scale. Locations on the logit scale where persons are represented with no corresponding item identifies gaps in the item difficulty continuum. The person measure score is another indicator of overall distribution. A person mean measure score location on the person item map, lower than the centralized item mean score of 50 indicates people in the sample were more able than the level of difficulty of the items. If the mean person location is higher (above 50), then the people in the sample were less able than the mean item difficulty.

#### Differential Item Analysis

To examine whether the scale items were used in the same way by all groups, a differential item functioning (DIF) analysis was performed on the remaining 23 items. DIF occurs when a characteristic other than the pragmatic language difficulty being assessed influences their rating on an item (Bond and Fox, 2015). For DIF analysis, the sample was categorized by age (5–8 years vs. 9–11 years), participant category (ADHD vs. Playmate vs. Control), ethnicity (European vs. Maori vs. Other ethnicities), gender (male vs. female), and pragmatic language difficulty (PLD vs. noPLD).

We were interested in these variables, (a) based on the current literature about the development of pragmatic language, and (b) given that POM is a measure of pragmatic language performance, we needed to establish if it could detect differences in performance of children with ADHD and possibly PLD, as we would expect this would impact their scores (Wu et al., 2017). Children with ADHD (Väisänen et al., 2014) and children with PLD (Ketelaars et al., 2010; Ryder and Leinonen, 2014) have been found to have poorer pragmatic language outcomes.

If, however, there was significant DIF on a large number of items based on comparing age, gender and ethnicity, this would be a concern, or at least warrant further research as it possibly indicates item bias. DIF based on age would only be expected with children younger than 5 years of age (Moreno-Manso et al., 2010; Hoffmann et al., 2013). The children in this study were older than 5 years of age. For the purposes of DIF analysis, two age groups (5–8 years vs. 9–11 years) were created by dividing the number of children into two groups that were of relatively equal size. We did initially attempt three age groups, but the number of children in the 5–6 years and the 10–11 years age categories were too small. If significant differences were detected in a large number of items when examining DIF between the two age groups that would indicate the need for further research to understand if it was the result of impact or bias. In terms of gender, previous research found that boys performed poorer than girls in pragmatic language outcomes (Ketelaars et al., 2010). Surprisingly, no research has been conducted that specifically explored pragmatic language in terms of cultural variations, even though differences in non-verbal communication across cultures have previously been acknowledged in research (Vrij et al., 1992; Dew and Ward, 1993).

Differential item functioning contrast is inspected when comparing groups and refers to the difference in difficulty of the item between both groups. When testing the hypothesis “this item has the same difficulty for two group,” DIF is noticeable when the DIF contrast, which is the reporting of effect size in Winsteps, is at least 0.5 logits with a *p*-value < 0.05, as statistical significance can be affected by sample size and the sample size may not be large enough to exclude the possibility of being accidental (Linacre, 2016a). When interpreting the directionality of DIF contrast values, if the logits are positive, then it indicates that the item was harder (lower scores) than expected. If the logits are negative, then it indicates that the item was easier (higher scores) than expected. In determining DIF when comparing more than two groups (i.e., participant category and ethnicity) with the hypothesis “this item has no overall DIF across all groups,” the chi-square statistic and *p*-value < 0.05 is used (Linacre, 2016a). Winsteps implements two DIF methods. Winsteps implements Mantel for complete or almost complete polytomous data, Mantel–Haenszel for uniform DIF analysis of complete or almost complete dichotomous data, and a logistic uniform DIF method for incomplete, especially sparse, data, which estimates the difference between the Rasch item difficulties for the two groups, holding everything else constant. Mantel/Mantel–Haenszel in Winsteps are (log-)odds estimators of DIF size and significance from cross-tabs of observations of the two groups and uses theta to stratify to overcome the limitation of needing complete data in its original form. Furthermore, Mantel/Mantel–Haenszel is suitable for our sample size as Mantel/Mantel–Haenszel does not require large sample (Guilera et al., 2013). Winsteps also implements a non-uniform DIF logistic technique and a graphical non-uniform DIF approach. For the DIF analysis conducted in this analysis we used the Mantel–Haenszel test for dichotomous variables and the Mantel test for polytomous variables as these methods are generally considered most authoritative (Linacre, 2016b).

## Results

The sample of 342 records from 108 children with ADHD and 108 typically developing playmates and 126 typically developing (TD) children were analyzed; 80.3% of the children with ADHD were male with a mean age of 8.9 years (*SD* = 1.4) and 75.2% of the playmates were males with a mean age of 8.4 years (*SD* = 1.9) and 78.7% in TD group were males with a mean age of 8.6 years (*SD* = 1.5). All children were from New Zealand with ethnicity representative of the New Zealand population. See Table 2 for details and other demographic information. Missing data were recorded for 9 (0.1%) out of 9,234 observations (27 items × 342 participants) which is negligible.

**Table 2:** Participant demographics.

Child and parent demographics	Children with ADHD (*n* = 108)	Playmates of children with ADHD (*n* = 108)	Typically developing children (*n* = 126)
Mean age (*SD*)	8.9 years (1.4)	8.4 (1.9)	8.6 years (1.5)
Percentage boys vs. girls	80.3%/19.7%	75.2%/24.8%	78.7%/21.3%
Ethnicity			
European	67.8% ^β^	65.2%	65.2%^β^
Maori	16.1% ^β^	16.1%	19.7%^β^
Other ethnicities	16.1% ^β^	18.7%	15.1%^β^
**CPRS-R subscale scores**			
Oppositional	70.4^∗^	56.9	50.6^§^
Cognitive Problems	72.5^∗^	51.4	49.5^§^
Anxious/Shy	58.9	50.3	50.8^§^
Perfectionism	56.1	49.8	49.3^§^
Social Problems	76.0^∗^	60.4	48.9^§^
Psycho-Somatic	64.4	49.8	50.6^§^
Emotional Labile	62.8	50.6	48.5^§^
Behavioral Problems	73.0^∗^	56.2	49.7^§^
**Primary carer’s highest level of education**			
Did not complete high school	13.4%	10.7%	19.1%
Completed high school	40.2%	39.3%	46.8%
Completed tertiary qualifications	46.4%	50.0%	34.1%
**Primary carer’s occupation**			
Jobs that do not require tertiary qualifications	63.4%	58.9%	75.4%
Jobs that do require tertiary qualification	36.6%	41.1%	24.6%

*^β^This is a close approximation of the current ethnic distribution of the New Zealand population estimate with Europeans making up 76.8%, Maori 14.9% and the remainder of ethnic groups 17.8% of the population, thus representative of the New Zealand population. *^§^ CPRS-R subscale mean scores are all below the clinical cut-off, (i.e., subscale scores > 70). ^∗^CPRS-R subscale mean scores above the clinical cut-off, (i.e., subscale scores > 70)*.*

### Rating Scale Validity

**Figure 1:**
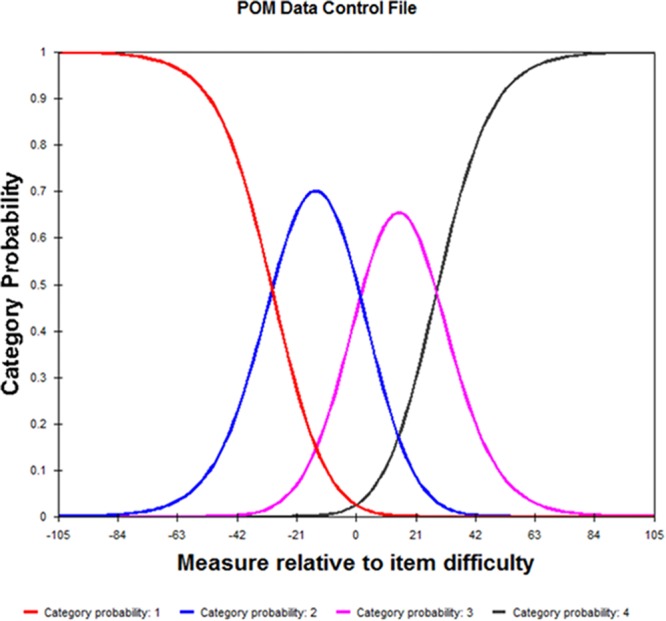
Rating scale validity.

The POM uses a 4-point (1–4) rating scale to rate the child’s performance from beginner to expert. For the overall instrument the probability of a category being observed was examined. The average measure scores increased monotonically, and the fit statistics were all in the acceptable range (MnSq = 0.7–1.4) resulting in four distinct, ordered categories (see Table 3 and Figure 1). When examining the Andrich thresholds which reflect the relative frequency of use of the categories, they were not disordered but all categories advanced by >5 logits (range 26.78–28.23 logits) indicating potential gaps in the measurement of the variable (i.e., in the category labels).

**Table 3:** Category function.

Category	*N*	%	Average measures	Infit MnSq	Outfit MnSq	Andrich thresholds
1	3171	34	-47.47	0.98	0.98	NONE
2	2702	29	-14.05	0.97	0.76	-29.68
3	2001	22	10.97	1.02	1.48	1.45
4	1351	15	37.45	1.07	1.06	28.23

*Missing data = 9; 0.001%.*

### Person and Item Fit Statistics

The summary infit and outfit statistics for item and person ability for the 27-item scale showed good fit to the model with a good item reliability estimate (0.99) and high person reliability (0.97). The PSI of 8.48 was well above the minimum of 3 required to separate people into distinct ability strata (see Table 4). Point biserial correlations were examined and all found to be in a positive direction indicating all items contribute to the overall construct.

**Table 4:** Item and person summary statistics.

								Infit		Outfit	
Analysis	Items	Item/ Person	Reliability	Separation	PSI^∗^	Mean Measure	Model *SE*	MnSq	Z-STD	MnSq	Z-STD
1	All 27 Items	Item	0.99	10.32	-	50.00	1.12	1.01	-0.1	1.03	-0.4
		Person	0.97	6.11	8.48	38.48	4.47	0.98	-0.1	0.96	-0.1
2	Self-regulation (SR) removed	Item	0.99	8.55	-	50.00	1.12	1.00	-0.2	0.97	-0.4
		Person	0.97	6.10	8.47	40.55	4.28	1.00	-0.1	0.98	-0.1
3	Creativity removed	Item	0.99	9.89	-	50.00	1.12	1.00	-0.1	1.14	-0.3
		Person	0.97	6.03	8.37	38.56	4.51	0.98	-0.1	0.97	-0.1
4	SR and Creativity removed	Item	0.98	7.95	-	50.00	1.13	1.00	-0.2	0.98	-0.3
		Person	0.97	6.02	8.36	40.35	4.29	1.00	-0.1	0.98	-0.1
5	SR, Creativity, Thinking style and Express feelings removed	Item	0.99	8.49	-	50.00	1.16	1.00	-0.2	0.97	-0.4
		Person	0.97	6.01	8.35	41.06	4.42	0.99	-0.1	0.97	-0.1
6	Creativity, Thinking style and Express feelings removed	Item	0.99	10.51	-	50.00	1.15	1.01	-0.1	1.19	-0.3
		Person	0.97	5.98	8.31	38.33	4.75	0.97	-0.1	0.95	-0.1
7	Creativity, Thinking style, Express feelings, and Request removed	Item	0.99	10.80	-	50.00	1.16	1.01	-0.1	1.21	-0.3
		Person	0.97	5.95	8.27	38.34	4.89	0.97	-0.1	0.95	0.0

*^∗^PSI, Person Separation Index/Strata; PSI = [4 × Person Separation + 1]/3. A Person Strata of, “3” (the minimum level to attain a reliability of 0.90) implies that three different levels of performance can be consistently identified by the test for samples like that tested*.

We then examined the summary fit statistics of the overall scale (all 27 items), where after we ran the analysis again, removing each of the outfitting items (all under-fitting), individually and then together to determine if this improved the overall fit to the model. The additional analyses were completed in the following sequence: (1) *self-regulation* only removed; (2) *creativity* only removed; (3) both *creativity* and *self-regulation* removed. This change in excluded items led to the removal of two further items: *thinking style* and *express feelings*. The analysis was then completed with the four items removed (*self-regulation*, *creativity*, *thinking style*, and *express feelings*). The *self-regulation* item was returned because the previous step had reduced item dimensionality and item separation was less. We then re-analyzed the data with the three mentioned items removed.

With each analysis the person and item reliability remained unchanged except when self-regulation and creativity were removed together it resulted in item reliability of 0.98 (from 0.99) but still well above the required 0.90 which confirms the hierarchy of the scale items. Item separation remained good, although it was reduced compared to all items when *self-regulation* and *creativity* were removed separately and together, and when removed with *thinking style* and *express feelings*. Person separation remained at approximately 6.0, well above the required level of 3 for all analyses and the person separation index remained high but did not improve with the removal of any items.

Following examination of the point biserial correlations that confirmed all items contributed to the overall construct we examined item misfit for all 27 items combined (see Table 5). We examined infit and outfit scores for contradictions and although there were more reported misfitting infit Z-STD scores than outfitting Z-STD scores, there were no contradictions in the direction of change. Underfit (MnSq > 1.4; Z-STD > 2) is the biggest threat to the measure because it can degrade the model as it occurs because of too much variation in the responses (Bond and Fox, 2015). Underfit of both infit and outfit scores was not observed for any item, but infit MnSq and Z-STD for *creativity* were both underfitting, as were the outfit MnSq and Z-STD for *self-regulation*, *thinking style*, and *express feelings*. More misfit was evident on infit and outfit Z-STD scores than MnSq with the Z-STD infit also underfitting for *self-regulation*, *thinking style*, *express feelings*, and *requests*. Overfit of the MnSq infit and outfit scores for *respond*, *environmental demands*, and *integrate communicative aspects* was also observed. Removing *self-regulation* resulted in a slight reduction in the underfit of *creativity* MnSQ, but this change slightly increased the outfit MnSq for *creativity* and *express feelings* without resulting in any improvements to model fit of other items. When *creativity* was removed there was underfit on all infit and outfit scores for *thinking style* and *express feelings*, and for *self-regulation* except infit MnSQ and there were no other significant or improved fit scores on the other items (Table 5). A similar outcome was observed for *thinking style* and *express feelings* when *creativity* and *self-regulation* were removed in the same analysis. Removing *creativity*, *thinking style* and *Express feelings*, with and without removing *Self- regulation* resulted in under fit for all scores for the *request* item and increased under infit Z-STD scores for *facial expressions* and *repair and review* (Table 5). In the final analysis we removed *creativity*, *thinking style*, *express feelings* and *requests* which resulted in an increase in the underfit of the *self-regulation* outfit MnSq (from 4.07 to 8.24) and an increase in the under fit of *self-regulation* infit and outfit Z-STD. This change also resulted in the underfit of *disagree* and *facial expression* infit Z-STD remaining and increased underfit of the *repair and review* and *initiate* Z-STD (Table 5). This solution was kept as it resulted in the best individual item fit that did not degrade person separation.

**Table 5:** Individual item fit statistics and principal component analysis for subscales.

	All 27 Items					Self-regulation removed					Creativity removed					SR and Creativity removed					SR, creativity, thinking style and express feelings removed					Creativity, thinking style and express feelings removed					Creativity, thinking style, rxpress feelings, and request removed				
	Infit		Outfit		PTM Corr.	Infit		Outfit		PTM Corr.	Infit		Outfit		PTM Corr.	Infit		Outfit		PTM Corr.	Infit		Outfit		PTM Corr.	Infit		Outfit		PTM Corr.	Infit		Outfit		PTM Corr.
Items	MnSq	Z-STD	MnSq	Z-STD		MnSq	Z-STD	MnSq	Z-STD		MnSq	Z-STD	MnSq	Z-STD		MnSq	Z-STD	MnSq	Z-STD		MnSq	Z-STD	MnSq	Z-STD		MnSq	Z-STD	MnSq	Z-STD		MnSq	Z-STD	MnSq	Z-STD	
Self-regulation	1.31	3.4	4.07	8.1	0.80	-	-	-	-	-	1.31	3.3	6.69	9.9	0.80	-	-	-	-	-	-	-	-	-	-	1.37	3.9	7.86	9.9	0.80	1.37	3.8	8.24	9.9	0.80
Thinking style	1.37	4.1	1.71	4.2	0.81	1.39	4.2	1.94	6.4	0.80	1.43	4.7	1.79	4.7	0.80	1.45	4.8	2.04	7.3	0.80	-	-	-	-	-	-	-	-	-	-	-	-	-	-	-
Express feelings	1.38	4.2	1.61	3.8	0.81	1.38	4.2	1.65	4.8	0.82	1.42	4.5	1.64	4.0	0.81	1.42	4.5	1.68	5.3	0.82	-	-	-	-	-	-	-	-	-	-	-	-	-	-	-
Creativity	1.59	5.4	1.36	1.2	0.73	1.57	5.2	1.46	1.5	0.73	-	-	-	-	-	-	-	-	-	-	-	-	-	-	-	-	-	-	-	-	-	-	-	-	-
Requests	1.29	3.2	1.24	1.5	0.82	1.27	3.0	1.29	2.2	0.82	1.32	3.5	1.28	1.7	0.82	1.30	3.4	1.34	2.6	0.83	1.40	4.3	1.54	4.2	0.82	1.42	4.5	1.42	2.3	0.82	-	-	-	-	-
Disagrees	1.06	0.7	1.17	1.1	0.84	1.05	0.7	1.24	1.8	0.84	1.08	0.9	1.20	1.2	0.84	1.07	0.9	1.27	2.0	0.84	1.13	1.5	1.38	3.0	0.85	1.13	1.6	1.31	1.7	0.84	1.17	2.0	1.34	1.9	0.84
Facial expression	1.16	1.9	1.05	0.4	0.85	1.19	2.2	1.13	1.1	0.85	1.17	2.0	1.06	0.5	0.85	1.20	2.3	1.14	1.3	0.85	1.24	2.7	1.21	2.0	0.85	1.21	2.4	1.10	0.8	0.85	1.22	2.5	1.10	0.8	0.85
Repair and review	1.13	1.4	0.80	-0.6	0.78	1.12	1.2	0.84	-0.5	0.78	1.17	1.8	0.83	-0.5	0.79	1.15	1.6	0.86	-0.4	0.79	1.22	2.2	0.95	-0.1	0.79	1.24	2.4	0.89	-0.3	0.79	1.29	2.8	0.93	-0.1	0.79
Initiate	1.05	0.6	0.93	-0.5	0.86	1.04	0.5	0.96	-0.3	0.86	1.08	1.0	0.96	-0.3	0.86	1.07	0.9	0.99	0.0	0.86	1.14	1.7	1.08	0.8	0.86	1.15	1.8	1.02	0.2	0.86	1.21	2.4	1.09	0.7	0.85
Body posture	1.05	0.6	0.91	-0.7	0.86	1.08	1.0	0.98	-0.1	0.86	1.05	0.6	0.91	-0.8	0.87	1.08	1.0	0.99	-0.1	0.87	1.09	1.1	1.03	0.3	0.87	1.05	0.7	0.92	-0.7	0.87	1.04	0.5	0.90	-0.8	0.88
Cooperation	1.04	0.5	0.92	-0.5	0.87	1.08	0.9	0.98	-0.1	0.87	1.04	0.5	0.92	-0.6	0.87	1.08	0.9	0.98	-0.1	0.87	1.07	0.9	0.99	0.0	0.88	1.02	0.3	0.91	-0.6	0.87	1.01	0.2	0.90	-0.7	0.88
Maintain and change	1.01	0.1	0.68	-1.0	0.78	1.01	0.1	0.72	-1.0	0.77	1.05	0.5	0.70	-0.9	0.78	1.05	0.5	0.74	-0.9	0.78	1.10	1.1	0.80	-0.6	0.78	1.10	1.0	0.74	-0.8	0.78	1.12	1.2	0.77	-0.7	0.79
Gestures	0.98	-0.2	0.97	-0.2	0.86	1.00	0.0	1.05	0.5	0.86	0.99	-0.1	0.98	-0.1	0.86	1.01	0.1	1.08	0.8	0.86	1.04	0.5	1.14	1.4	0.87	1.02	0.2	1.03	0.3	0.87	1.03	0.4	1.03	0.3	0.87
Communication content	0.97	-0.4	0.81	-1.2	0.86	0.97	-0.4	0.85	-1.2	0.86	1.00	0.1	0.85	-0.9	0.86	1.00	0.1	0.89	-0.8	0.86	1.10	1.2	1.02	0.2	0.85	1.10	1.1	0.94	-0.3	0.85	1.14	1.6	0.99	0.0	0.85
Engagement/Interaction	0.96	-0.5	0.92	-0.6	0.87	0.99	-0.1	0.98	-0.1	0.87	0.96	-0.5	0.90	-0.8	0.87	0.99	-0.1	0.97	-0.2	0.88	0.98	-0.2	0.99	-0.1	0.88	0.94	-0.7	0.90	-0.8	0.88	0.94	-0.7	0.89	-0.9	0.88
Perspective taking	0.95	-0.6	0.83	-1.3	0.87	0.95	-0.6	0.87	-1.1	0.87	0.95	-0.6	0.83	-1.3	0.87	0.95	-0.6	0.88	-1.2	0.87	0.95	-0.6	0.90	-1.0	0.87	0.94	-0.7	0.84	-1.2	0.87	0.94	-0.7	0.84	-1.2	0.88
Attention, plan, initiate	0.95	-0.6	0.84	-1.1	0.87	0.95	-0.7	0.88	-1.1	0.87	0.97	-0.3	0.88	-0.9	0.86	0.97	-0.3	0.92	-0.7	0.87	1.09	1.1	1.04	0.4	0.86	1.08	1.0	0.98	-0.1	0.86	1.13	1.6	1.06	0.5	0.86
Emotional attunement	0.94	-0.7	0.88	-0.9	0.87	0.96	-0.5	0.93	-0.6	0.87	0.94	-0.7	0.88	-1.0	0.87	0.96	-0.5	0.94	-0.6	0.87	0.95	-0.6	0.96	-0.4	0.88	0.93	-0.8	0.88	-0.9	0.88	0.92	-1.0	0.86	-1.1	0.88
Suggests	0.94	-0.7	0.90	-0.7	0.86	0.94	-0.7	1.00	0.0	0.86	0.95	-0.6	0.90	-0.7	0.87	0.95	-0.6	1.00	0.1	0.87	1.00	0.1	1.15	1.4	0.87	1.00	0.1	1.02	0.2	0.87	1.05	0.7	1.05	0.4	0.86
Select and introduce	0.93	-0.9	0.77	-1.5	0.86	0.92	-1.0	0.80	-1.7	0.86	0.95	-0.6	0.80	-1.3	0.86	0.94	-0.7	0.83	-1.5	0.86	1.01	0.1	0.91	-0.8	0.86	1.02	0.2	0.86	-0.9	0.86	1.06	0.7	0.89	-0.6	0.86
Contingency	0.92	-0.9	0.78	-1.2	0.85	0.92	-1.0	0.80	-1.5	0.85	0.94	-0.7	0.79	-1.1	0.85	0.93	-0.8	0.82	-1.3	0.85	0.98	-0.2	0.88	-0.9	0.85	0.99	-0.1	0.84	-0.8	0.85	1.02	0.3	0.90	-0.4	0.85
Conflict resolution	0.84	-2.0	0.73	-2.3	0.88	0.85	-1.9	0.77	-2.1	0.88	0.85	-1.9	0.75	-2.3	0.88	0.86	-1.7	0.79	-2.1	0.88	0.87	-1.6	0.82	-1.9	0.89	0.86	-1.8	0.76	-2.1	0.89	0.87	-1.7	0.79	-1.8	0.89
Distance	0.81	-2.5	0.75	-2.1	0.88	0.82	-2.3	0.80	-1.8	0.89	0.82	-2.3	0.77	-2.1	0.88	0.83	-2.2	0.82	-1.8	0.89	0.84	-2.1	0.86	-1.4	0.89	0.82	-2.3	0.79	-1.8	0.89	0.82	-2.3	0.79	-1.8	0.89
Assertion	0.79	-2.7	0.72	-2.4	0.89	0.80	-2.6	0.75	-2.3	0.89	0.81	-2.5	0.75	-2.3	0.89	0.81	-2.4	0.78	-2.3	0.89	0.89	-1.4	0.87	-1.3	0.89	0.88	-1.6	0.83	-1.5	0.88	0.90	-1.2	0.84	-1.3	0.88
Respond	0.69	-4.2	0.58	-2.9	0.88	0.68	-4.3	0.60	-3.5	0.88	0.70	-4.0	0.58	-2.9	0.89	0.68	-4.2	0.61	-3.5	0.89	0.71	-3.8	0.65	-3.3	0.89	0.72	-3.6	0.61	-2.5	0.89	0.74	-3.3	0.63	-2.3	0.89
Environ. demands	0.66	-4.6	0.56	-3.1	0.89	0.67	-4.5	0.60	-3.6	0.89	0.67	-4.4	0.58	-2.9	0.89	0.68	-4.3	0.62	-3.4	0.89	0.68	-4.2	0.64	-3.5	0.89	0.67	-4.4	0.59	-2.8	0.89	0.67	-4.5	0.58	-2.7	0.90
Integrate comm. aspects	0.50	-7.5	0.45	-5.5	0.92	0.51	-7.3	0.47	-5.7	0.92	0.50	-7.5	0.45	-5.8	0.92	0.51	-7.4	0.47	-6.4	0.92	0.50	-7.4	0.47	-6.7	0.92	0.50	-7.6	0.44	-5.8	0.92	0.49	-7.7	0.43	-5.9	0.92


### Dimensionality of the Scale

The dimensionality of the overall scale with all 27 items was examined using principal components analysis (PCA) of the residuals (Table 6). The Rasch dimension explained 77.1% with >40% considered a strong measurement of dimension. However, of the 77.1% explained variance, the person measures (60.8%) explained almost four times the variance explained by item measures (16.3%). The total raw unexplained variance (22.9%) had an eigenvalue of 27, resulting in the eigenvalue of first contrast being 5.49, which indicated the presence of a second dimension. The PCA of residuals divided the items into two groups related to verbal aspects of pragmatic language (items 1, 2, 3, 4, 5, 6, 16, 17, 23, 25, and 26) and non-verbal aspects of pragmatic language (consisting of items 7, 8, 9, 10, 11, 13, 14, 15, 20, 21, and 22). Based on the theoretical logic that pragmatic language consists of both verbal and non-verbal components, which is what we considered in constructing the scale to ensure all the features of the construct were being measured, this finding indicates that the items do form the one construct of pragmatic language. This finding is further supported by examining the disattenuated correlation between the person measures on the two sets of items (see Table 5). With the exception of one disattenuated correlations being 0.79 (item: Maintain and Change), the correlations for all other items exceeded 0.8, which indicates the items are unidimensional for practical purposes (i.e., a multidimensional analysis will produce effectively the same results as a unidimensional one).

**Table 6:** Standardized residual variance all 27 items.

Variance	Eigenvalue	Observed (%)	Expected (5)
Total raw variance in observations	118.16	100.0	100.0
Raw variance explained by measures	91.16	77.1	77.2
Raw variance explained by persons	71.86	60.8	60.9
Raw variance explained by items	19.27	16.3	16.3

**Figure 2:**
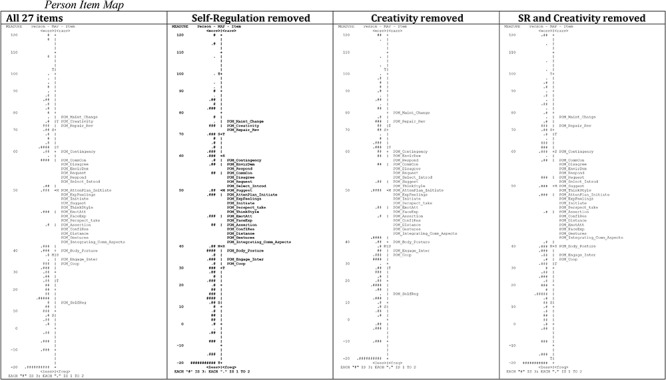
Person item map.

Second and subsequent contrasts were less than the 2 eigenvalue units required to indicate further dimensions (Table 7). This process was then repeated with the removal of *self-regulation*, and *creativity* (as the most misfitting items) separately, then both removed, and then in various combinations with *creativity*, *thinking style*, *express feelings*, and *request* without significant change and all models still indicating a second dimension (Table 7). As presented in Figure 2, the person-item maps show that few people were aligned with the missing items and that, although there were no redundant items, the addition of more easy and difficult items would improve the measure. The person-item dimensionality maps remained consistent, regardless of which items were removed.

**Table 7:** Standardized residual variance.

	Unexplained variance	Raw unexplained variance (total)	1st contrast	2nd contrast	3rd contrast	4th contrast	5th contrast
All 27 items	Eigen	27	5.49	2.21	2.16	1.61	1.31
	Obser.	22.90%	4.60%	1.90%	1.80%	1.40%	1.10%
	Exp.	100%	20.30%	8.20%	8.00%	6.00%	4.80%
Self-regulation removed	Eigen	26	5.47	2.22	2.14	1.6	1.3
	Obser.	25.00%	5.30%	2.10%	2.10%	1.50%	1.20%
	Exp.	100%	21.10%	8.50%	8.20%	6.20%	5.00%
Creativity removed	Eigen	26	5.48	2.2	2.12	1.58	1.22
	Obser.	23.10%	4.80%	2.00%	1.90%	1.40%	1.10%
	Exp.	100%	21.00%	8.40%	8.10%	6.10%	4.70%
SR and creativity removed	Eigen	25	5.45	2.19	2.12	1.58	1.2
	Obser.	25.60%	5.60%	2.20%	2.20%	1.60%	1.20%
	Exp.	100%	21.80%	8.80%	8.50%	6.30%	4.80%
SR, creativity, thinking style and express feelings removed	Eigen	23	5.26	2.13	1.95	1.51	1.15
	Obser.	25.20%	5.80%	2.30%	2.10%	1.70%	1.30%
	Exp.	100%	22.90%	9.20%	8.50%	6.60%	5.00%
Creativity, thinking style and express feelings removed	Eigen	24	5.26	2.15	1.93	1.5	1.16
	Obser.	22.20%	4.90%	2.00%	1.80%	1.40%	1.10%
	Exp.	100%	21.90%	8.90%	8.00%	6.30%	4.80%
Creativity, thinking style, express feelings, and request removed	Eigen	23	5.09	2.17	1.9	1.43	1.16
	Obser.	21.70%	4.80%	2.00%	1.80%	1.40%	1.10%
	Exp.	100%	22.10%	9.40%	8.10%	6.20%	5.10%

### Differential Item Analysis

The DIF analysis enabled examination of potential contrasting item-by-item profiles associated with: (a) ADHD, playmate or control, (b) age, (c) gender, (d) ethnicity, and (e) PLD vs. noPLD. The summary of the DIF analysis for the remaining 23 items is presented in Table 8 and revealed that participant category (ADHD vs. playmate vs. control) was the major factor in how items were used. DIF on the identified items indicated that children with ADHD scored lower than expected on items 7, 8, 9, 10, 11, 21, and 22, that is, children with ADHD found the items more difficult than expected. Children with ADHD found items 4, 16, 17, 23, 25, and 26 easier than expected. Based on category PLD vs. no pragmatic language difficulties (noPLD), none of the items had both a *p* < 0.05 and DIF contrast >0.5. DIF based on age of participants was only observed on items 8, 9, and 23, which were all harder than expected for the younger children. DIF based on gender was observed for three items with higher than expected scores (easier than expected) on items 12 and 23 for boys, and lower than expected scores (harder than expected) for item 13. DIF reported on items indicating it was easier for the European group to score on items 3, 6, and 17 and more difficult for them to score on the non-verbal items 7, 8, and 9.

**Table 8:** Summary DIF analysis.

	ADHD vs. Playmate vs. Control			Age 5–8; 9–11			Gender			Ethnicity			PLD vs. noPLD		
Items	Summary DIF Chi-squared	Prob.	DIF contrast (effect size)^&^	Mantel–Haenszel Prob.	Prob.	DIF Contrast (Effect Size)^∧^	Mantel–Haenszel Prob.	Prob.	DIF contrast (effect size)^#^	Summary DIF Chi-squared	Prob.	DIF contrast (effect size)^+^	Mantel–Haenszel Prob.	Prob.	DIF contrast (effect size)^$^
Select and introduce	6.3381	0.0411	-0.34	0.1331	0.7152	-	3.6017	0.0577	-0.70	5.7575	0.0550	-0.69	2.0000	0.1573	-0.35
Maintain and change	6.7402	0.0336	0.39	0.6547	0.4185	-0.33	0.3128	0.5759	-0.44	1.8615	0.3906	-0.47	-	-	-1.34
Contingency	4.9737	0.0816	-0.12	0.0067	0.9346	-0.06	0.5386	0.4630	-0.31	14.7734	0.0006^∗^	-1.05^∗^	0.5000	0.4795	-1.08
Initiate	14.6326	0.0006^∗^	-0.85^∗^	1.8634	0.1722	-0.15	1.0956	0.2952	-0.46	1.7792	0.4072	-0.37	1.4706	0.2253	-0.40
Respond	4.7235	0.0925	0.28	2.0158	0.1557	-0.21	3.2012	0.0736	-0.19	1.3469	0.5067	-0.32	1.0000	0.3173	-1.14
Repair and review	1.3696	0.5010	0.01	0.0868	0.7682	-0.29	0.0038	0.9510	-	7.3398	0.0249^∗^	-0.67^∗^	-	-	-1.76
Facial expression	8.5449	0.0136^∗^	0.52^∗^	0.8093	0.3683	0.34	0.4670	0.4944	0.29	13.9088	0.0009^∗^	0.99^∗^	1.0000	0.3173	0.21
Gestures	11.3712	0.0033^∗^	0.50^∗^	4.2619	0.0390^∗^	0.57^∗^	0.0444	0.8331	0.13	7.7211	0.0205^∗^	0.75^∗^	0.5000	0.4795	-
Body posture	18.7908	0.0001^∗^	1.10^∗^	6.6475	0.0099^∗^	0.69^∗^	0.0004	0.9836	0.14	13.1658	0.0013^∗^	0.82^∗^	0.2000	0.6547	-
Distance	9.2241	0.0097^∗^	0.72^∗^	2.3059	0.1289	-0.23	1.2993	0.2543	0.35	3.3993	0.1799	0.50^∗^	3.8571	0.0495	0.19
Emotional attunement	7.6309	0.0215^∗^	0.78^∗^	0.1635	0.6859	0.09	1.1590	0.2817	0.35	2.5818	0.2716	0.42	0.5000	0.4795	0.13
Self-regulation	0.5004	0.7785	-0.09	0.7271	0.3938	0.33	5.6478	0.0175^∗^	-0.81^∗^	0.6434	0.7240	-0.22	0.5000	0.4795	1.24
Perspective taking	1.5652	0.4537	0.23	0.9302	0.3348	-0.38	11.6599	0.0006^∗^	0.78^∗^	1.3782	0.4988	0.29	1.0000	0.3173	-0.03
Integrate comm. aspects	2.4041	0.2971	0.40	4.7833	0.0287	0.12	2.4845	0.1150	0.14	0.3273	0.8500	0.15	0.5000	0.4795	-1.10
Environ. demands	1.4732	0.4754	0.14	0.9558	0.3282	-0.09	3.5995	0.0578	0.36	1.7039	0.4230	0.36	2.0000	0.1573	-0.04
Attention, plan, initiate	19.9982	0.0000^∗^	-1.25^∗^	1.9012	0.1679	0.32	0.3486	0.5549	-0.25	4.9095	0.0842	-0.37	1.0000	0.3173	-0.16
Communication content	8.1563	0.0165^∗^	-0.81^∗^	0.3642	0.5462	-	0.1231	0.7257	-0.10	10.8586	0.004^∗^	-0.94^∗^	0.5000	0.4795	-0.26
Conflict resolution	4.6430	0.0963	-0.32	0.0008	0.9775	-0.27	2.7639	0.0964	0.31	2.8697	0.2349	-0.36	0.2000	0.6547	-0.12
Cooperation	11.2828	0.0034^∗^	0.83^∗^	2.3051	0.1290	-0.32	0.2578	0.6117	0.33	0.1184	0.9444	-0.09	0.2000	0.6547	0.28
Engagement/Interaction	16.2281	0.0003^∗^	1.04^∗^	3.9125	0.0479	-0.33	0.2908	0.5897	-0.06	4.7822	0.0898	0.26	0.2000	0.6547	0.04
Assertion	8.7447	0.0123^∗^	-0.72^∗^	4.9895	0.0255^∗^	0.61^∗^	6.4117	0.0113^∗^	-0.61^∗^	0.6146	0.7346	0.21	2.0000	0.1573	-0.14
Suggests	11.9110	0.0025^∗^	-0.99^∗^	3.6852	0.0549	-0.58	0.4662	0.4948	0.26	1.2276	0.5383	-0.15	1.0000	0.3173	-0.11
Disagrees	31.2661	<0.0001^∗^	-1.57^∗^	0.0004	0.9844	-0.14	0.5776	0.4473	0.25	1.0097	0.6011	0.21	0.5000	0.4795	0.57

*^&^ADHD vs. control (ADHD reference group); ^∧^5–8 vs. 9–11 (5–8 reference group); ^#^gender (male reference group); ^∗^Denotes items with p < 0.05 and Effect size (DIF contrast) > 0.5*.

*^+^Maori/Pacific Island vs. European (European reference group); ^$^PLD vs. noPLD (noPLD reference group); ^∗^Denotes items with p < 0.05 and Effect size (DIF contrast) > 0.5*.

## Discussion

In this study we aimed to evaluate the psychometric properties of the POM using Rasch analysis. Our first important finding was that with items *creativity, thinking style, express feelings*, and *requests* removed, the overall item and person reliability [the IRT equivalent of Cronbach’s Alpha/internal consistency (Mokkink et al., 2010; Bond and Fox, 2015)] of the POM was excellent, with the overall infit and outfit statistics within the required parameters. Additionally, the person and item separation indexes were within acceptable parameters. This indicates that the POM performs well in regard to separating children with different levels of pragmatic language skills into four distinct groups (i.e., scores 1–4 indicating skill levels of beginner, advanced beginner, competent or expert) (Bond and Fox, 2015).

Our next important finding is related to the dimensionality of the POM and the removal of misfitting items *creativity, thinking style, express feelings*, and *requests*. Using Rasch analysis, dimensionality reflects the structural validity of the POM (Baylor et al., 2011; Bond and Fox, 2015). The low percentage of overall unexplained variance across the 27 items (22.9%) supports the finding that the POM is a unidimensional construct with good structural validity. However, our analyses indicated that there were some items that did not contribute to toward the overall construct. When items *creativity, thinking style, expresses feelings*, and *requests* were removed, the unexplained variance reduced to 21.7%.

We suspect these items may not have contributed to the construct as they are in part subsumed within items in the areas of *Introduction and Responsiveness* and *Social-Emotional Attunement* (Campbell et al., 2016). For instance, to *express feelings* appropriately and successfully continue a communicative interaction one needs to be able to: (1) regulate their own thinking, emotions and behavior (item: *self-regulation*); (2) be aware of and respond to another’s emotional needs (item: *emotional attunement*); and (3) consider and integrate another’s viewpoint/emotion (item: *perspective taking*). These items sit under the element of Social-Emotional Attunement in the first version of the POM.

Similarly, to think and articulate abstract and complex ideas (item: *thinking style*) and interpret, connect, and express ideas in versatile ways (item: *creativity*) and request explanations or more information (item: *requests*) to effectively continue a communicative interaction, one must be able to use skills in the area of *Introduction and Responsiveness.* These skills include the ability to *select and introduce* and *maintain and change* conversational topics. In order to be able to *respond* to communication from another and *repair or review* conversation when a breakdown occurs, one must also be able to successfully *request* information from their communication partner (Sehley and Snow, 1992).

At an individual item level, after removing items *creativity*, *thinking style*, *express feelings*, and *requests*, the MnSq fit statistics of most items were within acceptable parameters. However, the Z-STD fit statistic of five items (*self-regulation, disagrees, facial expression, repair and review*, and *initiates*) were outside of the expected parameters. *Self-regulation* was the most problematic item, falling outside the acceptable parameters for both infit and outfit statistics. This is not a surprising finding given the complex dynamic nature of self-regulation, which children are developing into adolescence (Raffaelli et al., 2005; McClelland et al., 2015). Additionally, self-regulation relates to both children’s social and emotional skills, which have been notoriously hard to define and measure (McClelland and Cameron, 2012; Jones et al., 2016). Conceptualizing and measuring self-regulation and other social-emotional skills has been described as a complex task as these skills are categorized broadly, with each containing a set of more delineated skills. In addition to measurement of self-regulation being hindered by lack of conceptual clarity, there is also existing debate as to the underlying components contributing to one’s capacity to self-regulate (McClelland and Cameron, 2012; Campbell et al., 2016; Jones et al., 2016).

Self-regulation was also problematic regarding the person-item dimensionality map as it was relatively easy in comparison to the rest of the POM items, despite it being a skill that is known to be notoriously complex from both clinical and conceptual viewpoints (McClelland and Cameron, 2012; Jones et al., 2016). The findings pointed to the need to change rather than drop the item. Furthermore, our findings indicated that there was evidence for both easy and hard items and people alignment alongside the items. Further, there was no evidence of item redundancy, with no items occurring at the same level.

Differential item functioning analyses were conducted in relation to participant group (ADHD, playmate, and control), age category (5–8 years or 9–11 years), gender, ethnicity and PLD vs. noPLD. Our finding that 13 of 23 items were significantly different in relation to clinical group is supported by previous research that found children with ADHD experience difficulty with pragmatic language skills compared to their peers (Kim and Kaiser, 2000; Bignell and Cain, 2007). Of interest is that many of the items that were more difficult were the non-verbal pragmatic language items and co-operation and engagement. Items that were easier included being assertive, making suggestions and disagreeing and initiation and, surprisingly, they found it easier than expected to manage communication content and the item ‘attend, plan and initiate.’ It is not surprising to see results that suggest that children with ADHD have more PLD compared to typically developing peers. In earlier studies, children with ADHD experienced more PLD than typically developing peers when using the Children’s Communication Checklist - Second Edition (CCC-2) (Väisänen et al., 2014), the Test of Pragmatic Language - Second Edition (TOPL-2), and the Comprehensive Assessment of Spoken Language (CASL) (Staikova et al., 2013). However, the exact nature of the pragmatic difficulties was reported at an aggregate level only, thus not allowing for a more nuanced explanation of differences. As such, this mix of DIF on items indicate that more research is required to understand whether the DIF is related to impact (i.e., the variable skills of children with ADHD compared to the controls), or bias in the items.

For children with PLD vs. noPLD, none of the items showed significant DIF. A possible explanation that there were no DIF for the PLD variable could be how the variable was derived. Children, regardless of diagnosis, who score 1.5 standard deviations below the overall mean measure score were categorized as PLD and those who score above the cut off score were categorized as noPLD. The cut off score of 1.5 standard deviations below the overall mean measure score may have been too conservative to truly identify children with PLD. In future development of the POM, a new cut-off point that is at least two standard deviations below the mean should be considered with a larger sample size.

Very few items were significantly different in relation to age (three items) or gender (three items). Two of the significantly different items for gender (*self-regulation* and *perspective taking*) are consistent with research that found school-aged girls demonstrated higher skills than boys on objective and teacher reported measures (Matthews et al., 2009). In terms of gender, boys have been reported to perform significantly lower than girls on the CCC-2 (Ketelaars et al., 2010). However, given that DIF was only observed for three items, further research is required to determine if this is related to inherent differences in the groups (impact) or bias (i.e., that the items were working differently based on gender).

In relation to ethnicity (European, Maori or other), only 7 items were found to be significantly different between children. This finding indicates the POM may have good cross-cultural validity. However, it is important to note that the majority of the sample was European and children were only categorized into three separate ethic groups. Testing the psychometric properties of the POM on a sample of children from a broader range of ethnic groups is therefore a required direction for further research. Given the relative homogeneity and the size of this sample, further research with larger sample sizes and different clinical populations is required to determine if DIF should be interpreted as impact or bias.

The POM is a measure of pragmatic language performance and it needs to differentiate on some items across non-verbal and verbal elements of pragmatic language performance. Further research is required to understand whether DIF observed (*p* < 0.05 and DIF contrast ≥0.5 logits) on the small number of age, gender and ethnicity items is due to impact or bias. Given the age-related development of pragmatic language skills, difference would be expected between very young children who would not be expected to have developed the pragmatic language skills compared with older children (Hoffmann et al., 2013). However, you would not expect to find DIF between children aged 5–11 years, which was the age range of the study participants (Moreno-Manso et al., 2010; Hoffmann et al., 2013). When viewed holistically, aside from ADHD as a diagnostic category, none of the other variables that were evaluated showed significant DIF across various items. This indicates the POM is consistent in measuring the underlying construct of pragmatic language, regardless of the child’s age, gender, ethnicity and PLD status.

### Resulting Changes to the POM

Our findings indicate that several changes to the POM are required. First, we needed to remove the four misfitting items *creativity, thinking style*, *express feelings*, and *requests*. Second, the standardized residual loadings indicated the presence of two dimensions. However, a more precise interpretation is that we are observing the covariance that is expected in such a complex construct (Linacre, 2016a). The two groups of items comprise verbal and non-verbal aspects of pragmatic language which are of equal weighting, that is, two attributes that contribute equally to the same construct (pragmatic language). Therefore, we interpret the findings that the POM is a unidimensional construct that comprises two elements named: Pragmatics Observational Measure Verbal Communication Element and Pragmatics Observational Measure Non-verbal Communication Element. These two elements replace the previous five elements we had in the POM that were based on theoretical constructs. This delineation of items allows for the calculation of a non-verbal and verbal pragmatic language score for children, as well as an overall pragmatic language measure score. Third, because of the aforementioned change, revisions to the four skill-level descriptions of item 23 (*Assertion*) were required. The previous description consisted of both verbal and non-verbal behaviors, and the revised description now only includes behaviors of verbal assertion.

The fourth and final change involved revising the description of the item *self-regulation* at each of the four skill levels in the POM. The revised item now includes a more detailed description at each skill level to better capture the complex delineation of skills that comprise self-regulation that is based on a review of research into the construct of self-regulation (McClelland and Cameron, 2012; Jones et al., 2016).

### Future Directions for Research

Based on the findings from this study, there are several important areas of research for the continued development of the POM. The first being testing the reliability and validity of the measure on children in broader diagnostic and ethnic groups. This should also include a greater number of typically developing females, so gender differences can be further examined (Mokkink et al., 2010). The revised items *Assertion* and *self-regulation* necessitates current raters to be provided with additional training in the observation of verbal assertion and self-regulation skills, using the revised descriptors.

Another important area for further development is making the POM accessible to allied health professionals, such as speech pathologists, occupational therapists, and psychologists who routinely work with children with PLD (Duncan and Murray, 2012). While the routine use of outcome measures has been mandated within the practice of allied health professionals for over two decades, barriers to the use of outcome measures still exists (Duncan and Murray, 2012). Thus, a training package for clinicians in the use of the POM and scoring and interpreting the scores should be carefully considered at both an individual and organizational level. It is likely that such a training package will need to also address: (1) the organization’s need to provide appropriate training, administrative support and allocation of resources to clinicians; (2) clearly highlight to clinicians the relevance and clinical applicability of the measure to direct client care; (3) address barriers around the time taken to compete the measure, considering the institutional restrictions which impact how much time a clinician spends with each patient/client; and (4) ensure the development of clinician knowledge of pragmatic language and strategies for facilitating the pragmatic language outcomes of children (Duncan and Murray, 2012).

### Limitations

The psychometric properties of the POM-2 need to be assessed in other clinical groups likely to have pragmatic language difficulties, including autism spectrum disorders and children diagnosed with Social Communication Disorder. The use of a single rater is also a limitation to be considered when interpreting the results. The use of multiple raters could impact POM-2 psychometric properties such as differential item functioning and dimensionality. Future research needs to, (a) investigate the responsiveness of the POM-2, (b) consider re-evaluating the POM-2 with additional items that are easier and more difficult to rate, (c) assess test-retest reliability of the POM-2 and (d) assess the POM with multiple raters and examine rater effect and severity using a multifaceted Rasch model (Linacre, 2018).

## Author Contributions

RC, NM, SW-G, RS, LP, and AJ contributed to the conceptual content of the manuscript. RC and AJ performed the statistical analysis. RC wrote the first draft of the manuscript. All authors contributed to manuscript revision, read and approved the submitted version.

## Conflict of Interest Statement

The authors declare that the research was conducted in the absence of any commercial or financial relationships that could be construed as a potential conflict of interest.
